# Engineering platelets for tumour targeting

**DOI:** 10.18632/aging.101014

**Published:** 2016-08-07

**Authors:** Gerard Marriott

**Affiliations:** Department of Bioengineering, UC-Berkeley, CA 94720, USA

**Keywords:** repurposed platelets, cancer, in vivo imaging, drug delivery, platelet-rich therapy

Human platelets are small (2μm), anucleate, discoid-shaped living cells that play essential roles in vascular haemostasis, thrombosis and inflammation [1]. While platelets are primarily confined to blood vessels, they may also accumulate in tumour microenvironments where they modulate functions of both tumour and immune cells [1]. It is known that platelets gain access to tumour microenvironments by passive diffusion across leaky capillaries, and through associations with activated immune cells [1]. Given this privileged access, one could, by imparting human platelets with new molecular functions, repurpose them as vehicles for tumour cell-targeting, in vivo imaging and delivery of therapeutic agents. This idea however, is undermined by the fact that human platelets aggregate easily in response to the types of chemical and physical manipulations envisaged for their repurposing. We have found a simple solution to address this issue that involves incubating freshly acquired human platelets with a membrane-permeable natural product drug that inhibits actin polymerization or blocks platelet activation [2]. Moreover, we have developed robust protocols to introduce aggregation incompetent human platelets with surface-coupled antibodies against tumor biomarkers, and with cytosol-trapped small molecule probes for magnetic resonance- or near infra-red fluorescence (NIRF) imaging, and also with cytotoxins for tumour therapy. The reactions used to repurpose the platelets as vehicles for in vivo imaging and therapy are typically complete within 60-minutes, and may be conducted within a custom-built automated device. Significantly, we have shown that the repurposed platelets engage in specific interactions with tumour cells in xenotransplants where they can generate an intense Cy7-fluorescence signal, allowing for high-contrast NIRF imaging of early stage tumours [2].

One might ask what other advantages do human platelets have over artificial nanoparticles in targeting tumours aside from their privileged access to tumour microenvironments [2.^3^]. This question is best answered by highlighting functional differences between the two types of vehicles [2-4]. First, most nanoparticles are rapidly removed from circulation, and in addition, the sudden appearance of cytotoxin may trigger apoptosis in the scavenging macrophages and liver cells. Repurposed platelets on the other hand, owing to their large size, can only be scavenged by Kupffer cells in the liver and spleen. Furthermore, platelets circulate for up to 9-days, during which time they are more likely to gain access to deep-seated microenvironments in a tumour, while pacing the exposure of Kupffer cells to the cytotoxin. Second, with a diameter of ~2μm, platelets can accommodate more than 100-fold more copies of a surface-coupled antibody, and a thousand-fold larger cargo of internalised imaging probes and/or cytotoxin molecules [2]. Third, human platelets, unlike tumour-targeting nanoparticles, are living and breathing cells that harbour mitochondria and functional membrane-bound proteins including transporters and esterases that allow the platelets to retain actively exogenous small molecule probes and cytotoxins [2].

If platelets obtained from the blood bank are injected into a patient under treatment, there may be a risk of rejection or infection due to contaminations from red blood cells or bacteria. However, these problems can be minimized if the patient's own platelets are used and the injection is performed shortly after drawing blood. This is the case for platelet-rich therapy (PRT) [5], where the platelets are isolated from the patient's blood, concentrated, and then injected at treatment sites within an hour. PRT can also be performed in a non-hospital facility by a doctor or qualified nurse [5]. Looking ahead, we see opportunities to develop platelet-based personalized medicine, by borrowing the practice of PRT and integrating with the disease-specific repurposing of platelets, for the diagnoses and treatment of cancer or neurologic and cardiovascular diseases.

## References

[1] Weyrich AS, Zimmerman GA (2004). Trends Immunol.

[2] Dai L (2016). Oncotarget.

[3] Lane LA (2015). Ann. Rev Phys Chem..

[4] Hu CM (2015). Nature.

[5] Foster TE (2009). The American journal of sports medicine.

